# Technologies Applied to the Mental Health Care of Pregnant Women: A Systematic Literature Review

**DOI:** 10.1055/s-0043-1768458

**Published:** 2023-04-27

**Authors:** Laís Lage de Carvalho, Júlia Magna da Silva Teixeira, Roberto José Gervásio Unger, Vivian Genaro Motti, Giovanni Marcos Lovisi, Fabiane Rossi dos Santos Grincenkov

**Affiliations:** 1 Centro Universo Juiz de Fora, Curso de Psicologia, Juiz de Fora, MG, Brazil; 2 Universidade Federal de Juiz de Fora, Juiz de Fora, MG, Brazil; 3 Universidade Federal do Rio de Janeiro, Rio de Janeiro, RJ, Brazil; 4 George Mason University, Information Sciences and Technology, Fairfax, VA, United States

**Keywords:** Pregnancy, Telemedicine, Mental health, Prenatal care, Pregnancy complications, Gravidez, Telemedicina, Saúde mental, Cuidado pré-natal, Complicações da gravidez

## Abstract

**Objective:** This article aims to review the literature regarding the use of technologies to promote mental health for pregnant women. We seek to: understand the strategies that pregnant women use for mental health care. Also, we investigate the existence of scientific evidence that validates such practices.

**Methods:** This study follows the PRISMA guidelines for systematic reviews. We analyze 27 studies published between 2012 and 2019. We include publications in Portuguese, English, and Spanish.

**Results:** The results revealed several different possibilities to use technology, including the use of text messages and mobile applications on smartphones. Mobile applications are the most commonly used approaches (22.5%). Regarding the strategies used, cognitive-behavioral approaches, including mood checks, relaxation exercises, and psychoeducation comprised 44.12% of the content.

**Conclusion:** There is a need for further investigation and research and development efforts in this field to better understand the possibilities of intervention in mental health in the digital age.

## Resumo

**Objetivo:** Este artigo objetiva revisar a literatura quanto ao uso das tecnologias como promotoras de saúde mental de gestantes. Desta forma, compreender quais são as estratégias utilizadas no cuidado da saúde mental dessas mulheres, assim como verificar se há evidências científicas que justifiquem a implementação dessas práticas.

**Métodos:** Este estudo segue o protocolo PRISMA para revisões sistemáticas de 27 estudos publicados em 2012-2019, incluindo publicações em português, inglês e espanhol.

**Resultados:** Os resultados revelaram diferentes possibilidades de utilização da tecnologia, sendo o uso de mensagens de texto e de aplicativos em smartphones mais os utilizados (22,5%). No que se refere às ferramentas utilizadas, estratégias cognitivo-comportamentais, tais como verificação do humor, exercícios de relaxamento e psicoeducação compreenderam 44,12% do conteúdo.

**Conclusão:** Verifica-se a necessidade de mais investimentos nessa área para que se possa compreender as possibilidades de intervenção em saúde mental na era digital.

## Introduction

Pregnancy is an important period in a woman's life being characterized by substantial changes. Specifically, pregnancy leads to several changes in the women's body, affecting organs, systems, and cycles, which impact physical, hormonal, and psychosocial aspects of a woman's life. Such changes also have a direct impact on the mental health of pregnant women. 1 2 3 4 Therefore, pregnancy demands specialized care for women's health. Such care is particularly important when risk factors exist.

Health outcomes for mental health during pregnancy and postpartum are linked to numerous risk factors. Risk factors include the history of mental illness, unwanted pregnancy, use of alcohol or illicit substances, low education level, financial burden, and unemployment. In addition to that hormonal changes may also result in symptoms related to anxiety and depression, which can lead to psychological suffering. 5 6 7 Among protective factors that reduce risk, we can highlight prenatal care, education, and social support. 3 8

Among protective factors, we also remark the importance of activities that promote mental health during pregnancy. Such activities encompass multidisciplinary expertise, 9 10 aiming to reduce risk factors while augmenting protective ones. 11 More specifically, mental health interventions train women by offering support, educative information, and preparation during their pregnancy, aiding pregnant women not only to recognize and express their emotions, 12 but also to learn strategies to cope with emotions.

There are several ways to provide care and support for pregnant women, including the use of technologies, mobile devices, smartphones, as well as monitoring equipment, personal assistants, and other wireless devices. 13 14 15 16 The usage of technology to promote health, defined by the World Health Organization (WHO) as mobile health (mHealth) *,* is innovative and seeks to promote prenatal care. 17 The definition of mobile health (mHealth) is a “public health practice supported by mobile devices, such as mobile phones, patient monitoring devices, personal digital assistants (PDAs), and other wireless devices” (p. 6). 18 Technology usage for healthcare in general contributes to improve information dissemination. It also facilitates healthcare delivery, and coordination among specialized services. Lastly, it also involves pregnant women more closely and actively in their own healthcare practices. 18 19

According to recent literature reviews on mHealth during pregnancy, technological interventions that support healthcare strategies result in positive outcomes, for example by accelerating the access to health services, 17 reducing patients' concerns, reducing symptoms of anxiety and depression, 20 and managing pregnancy risks when those exist (e.g., in case of gestational diabetes). 15

The type of technology used varies, ranging from web-based informative programs to remote monitoring, telehealth, mobile care, and online psychotherapy. 21 22 23 24 25 Taking into account that child-bearing age women are frequent users of technology, and the growing need to investigate evidence-based approaches around this topic, 22 this paper aims to review the literature regarding the use of mHealth technologies to promote mental health for women during pregnancy and postpartum. Our goal is to better understand what the strategies are employed in mental health care for these women, as well as to identify the scientific evidence that validate such practices.

## Methods

We conducted a systematic literature review seeking to analyze research studies previously published, and to systematically understand the topic of interest. 26 We aim to synthesize previous studies, and to obtain a unified view of research findings by following a scientific and systematic process. Therefore, we structured our systematic review based on a scientific investigation that includes several decision points regarding the inclusion and exclusion of research studies according to a pre-defined set of criteria. Our approach also seeks to assess the validity of the findings obtained with the literature review. 27 Specifically, the search was carried out following conventional scientific standards as outlined in the Preferred Reporting Items for Systematic Review and Meta-Analysis Protocols (PRISMA). 28

The analysis of the literature included six steps: (1) identification of the topic of interest and research questions; (2) definition of the inclusion and exclusion criteria; (3) gathering and selection of related work; (4) categorization of selected work; (5) analysis and interpretation of the results; and (6) presentation of a synthesis of the topic investigated. The inclusion criteria defined by the authors are papers published in Portuguese, English, or Spanish whose document is available online; studies published and indexed in the following bases Lilacs, Medline/PubMed, PsycINFO, Scielo, Scopus and Web of Science; work related to the topic of interest (use of mHealth technology to promote mental health for pregnant women). The papers were selected for analysis regardless of their publication dates. For analysis, we excluded papers that present results related to babies, children, or postpartum.

The descriptors selected were also pre-defined based on terms presented in the Descriptors in Health Science ( *Descritores em Ciências da Saúde* - DeCS) and Medical Subject Headings (MeSH). The search took place in January 2020, using crossed- reference descriptors and Boolean operations including terms in Portuguese, English, and Spanish. The descriptors used were: 1) Gestantes " *Pregnant Women* " “ *Mujeres Embarazadas* ”, 2) Complicações da gravidez " *Pregnancy Complications* " “ *Complicaciones del Embarazo* ”, 3) Transtornos mentais " *Mental Disorders* " “ *Trastornos Mentales* ”, 4) Ansiedade " *Anxiety* " “ *Ansiedad* ”, 5) Depressão " *Depression* " “ *Depresión* ”, 6) Estresse psicológico “ *Stress, Psychological* ” “ *Estrés Psicológico* ”, 7) Cuidado pré-natal “ *Prenatal care* ” “ *Atención Prenatal* ”, 8) Sistemas de apoio psicossocial “ *Psychosocial Support Systems* ” “ *Sistemas de Apoyo Psicosocial* ”, 9) Apoio social “ *Social Support* ” “ *Apoyo Social* ”, 10) Fatores de risco “ *Risk Factors* ” “ *Factores de Riesgo* ”, 11) Telefone celular “ *Cell Phone* ” “ *Teléfono Celular* ” e 12) Telemedicina “ *Telemedicine* ” “ *Telemedicina* ”. The descriptors mentioned above were distributed in 15 search strings, defined as follows ( Chart 1 ).

**Table:** Chart 1 Search strategy

Search strings
Gestantes AND Complicações da gravidez AND transtornos mentais AND Cuidado pré-natal AND Sistemas de apoio psicossocial AND Apoio social AND Fatores de risco AND Telefone celular
Pregnant women AND Pregnancy complications AND Mental disorders AND Prenatal care AND Psychosocial support systems AND social support AND Risk factors AND Telemedicine
Pregnant women AND Pregnancy complications AND Anxiety AND Prenatal care AND Psychosocial support systems AND social support AND Risk factors AND Cell phone
Pregnant women AND Pregnancy complications AND Anxiety AND Prenatal care AND Psychosocial support systems AND social support AND Risk factors AND Telemedicine
Pregnant women AND Pregnancy complications AND depression AND Prenatal care AND Psychosocial support systems AND social support AND Risk factors AND Cell phone
Pregnant women AND Pregnancy complications AND depression AND Prenatal care AND Psychosocial support systems AND social support AND Risk factors AND Telemedicine
Pregnant women AND Pregnancy complications AND Stress psychological AND Prenatal care AND Psychosocial support systems AND social support AND Risk factors AND Cell phone
Pregnant women AND Pregnancy complications AND Stress psychological AND Prenatal care AND Psychosocial support systems AND social support AND Risk factors AND Telemedicine
Pregnant women AND Mental disorders AND Prenatal care AND Psychosocial support systems AND Risk factors AND Cell phone
Pregnant women AND Mental disorders AND Cell phone
Pregnant women AND Mental disorders AND Telemedicine
Pregnant women AND Anxiety AND Cell phone
Pregnant women AND Anxiety AND Telemedicine
Pregnant women AND depression AND Cell phone
Pregnant women AND depression AND Telemedicine

The advanced search resulted in 1,010 papers. Two researchers used the tool EndNote Web to organize the data and followed the protocol PRISMA to proceed with the review. 28 Also, the authors used a validated technique 28 (PRISMA) to extract data directly from the primary source and as such select relevant information from the publications gathered for further analysis. The data analysis relied on descriptive statistics, and content analysis. Content analysis encompasses a series of analysis techniques for communication. It employs systematic procedures and objectives, aiming to describe the information obtained. 29 After reading all articles, the authors grouped the information in categories by completing a categorical content analysis. The review was not registered.

## Results

**Fig. 1:**
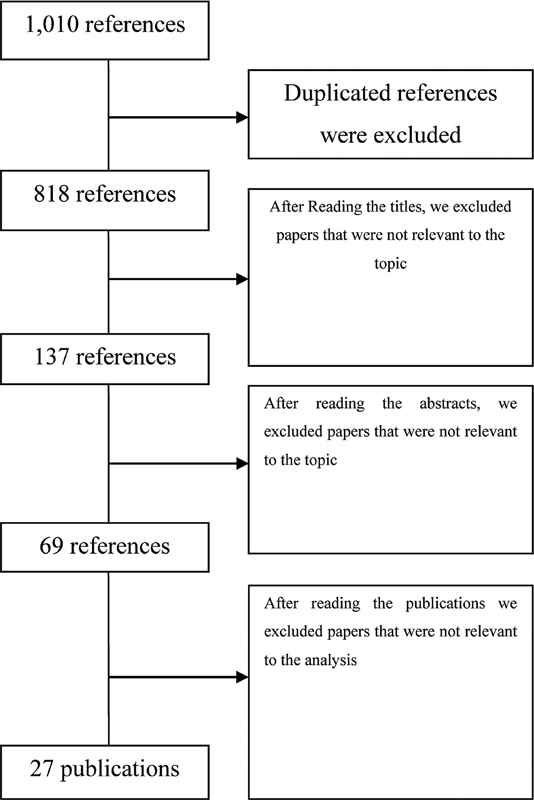
Selection of related work through the application of pre-defined exclusion and inclusion criteria.

Initially, we obtained 1,010 references from the databases used. After refining the search to identify duplicated work, we excluded 192 studies. Then, we discarded 681 articles, because the title of the work was not relevant to the review according to our inclusion criteria. After carefully reading the abstracts, we excluded 68 articles, and 69 publications remained for analysis. Lastly, after searching for the full documents, we excluded 42 studies because after reading the full papers of the studies we noticed that those did not meet our inclusion criteria. Thus, we obtained 27 papers for in-depth analysis, as illustrated in the Figure 1 .

In our analysis, we identified the journals where the articles were published, to identify those that most frequently publish articles related to the topic. We noticed that the journals *Annual Review of Cybertherapy and Telemedicine, BMC Psychiatry, Journal of Medical Internet Research* and *PLOS ONE* published each two papers related to the topic. We also noticed that, based on the research field of the first author, 29.6% of the articles were led by investigators from Health Sciences, followed by Psychology (18.5%) and Psychiatry (18.5%), as listed in Table 1 . Among the 27 papers analyzed, 7 (25.9%) reported literature reviews, thus we advance the state-of-the-art by updating the current analysis of the literature in the domain.

**Table 1:** Research fields from the first author

Research Field	Count	Percentage
Health Sciences	8	29.6
Psychology	5	18.5
Psychiatry	5	18.5
Medical Sciences	4	14.8
Nursing	2	7.4
Anthropology	1	3.7
Social Sciences	1	3.7
Health, arts, and design	1	3.7
Total	27	100.00

**Fig. 2:**
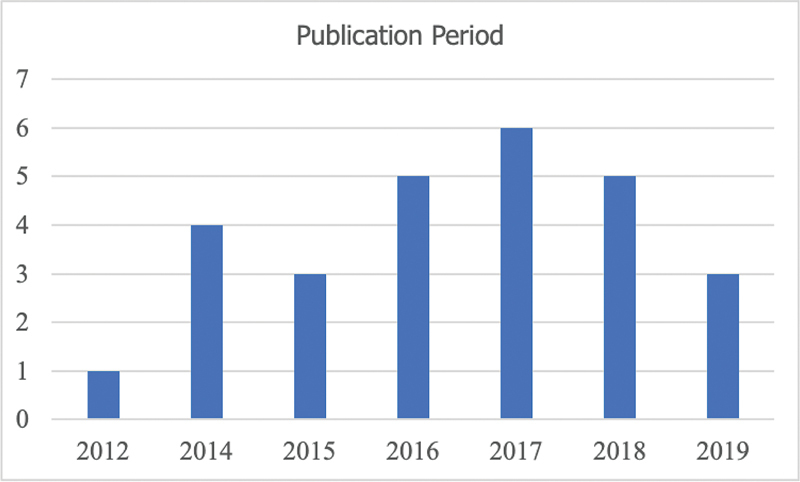
Publication period.

Our search did not return results from papers published in Portuguese, reporting studies carried out in Brazil. Moreover, the United States is the country with the largest number of studies in the domain – eight in total (29.6%), followed by Italy – with four (14.8%). Australia, Canada, United Kingdom and The Netherlands that conducted two studies each (7.4%). Also, there was only one study reported from Norway, Spain, Sweden, Germany, and Nigeria (3.7%). Lastly, the papers analyzed were published after 2012, as Figure 2 shows.

Regarding the type of technology used, the use of Short Message Service (SMS) and applications for mobile phones were commonly found (9 publications each – 22.5%), followed by phone calls and telehealth in general (5 publications each – 12.5%). One result returned for strategies related to audio recordings, online therapy (self-paced), video usage, voice-over-PowerPoint, and website (2.5%). Two studies reported voice messages and computer-assisted therapy (5.0%). Two publications did not specify the type of technology used (5.0%). Chart 2 summarizes the main aspects of each analyzed text, namely: main author, year of publication, study design, sample size and characteristics of the technology presented.

**Table:** Chart 2 Characteristics of the technologies used for pregnant women care

Author Year	Study design	Study population	Sample size	Technology features
Carissoli et al. (2017) 30	Longitudinal	Italian women, primiparous and in the third trimester of pregnancy	78	BenEssere Mamma is a mobile app consisting of a four-week pregnancy wellness self-help program. The app includes an experimental area with a set of daily relaxation exercises and guided imagery exercises and an emotional awareness area with a mood diary in which the user can record notes about her emotional state, thoughts and events that happen during the session.
Carissoli et al. (2016) 13	Longitudinal	women who participate in an aquatic gymnastics course for pregnant women, being over 18 years of age in low-risk pregnancies.	12	BenEssere Mamma is a mobile app consisting of a four-week pregnancy wellness self-help program. The app includes an experimental area with a set of daily relaxation exercises and guided imagery exercises and an emotional awareness area.
Davis et al. (2014) 14	Longitudinal	Study 1 and 2: Pregnant women attending appointments at the Obstetrics and Gynecology Clinic at the University of Kansas Medical Center	Study 1: 68; Study 2: 5	daily text messages (a total of five text messages per week, successively focused on one of three health topics) and three 20-minute voice-over-PowerPoints
Oyeyemi and Wynn (2015) 17	Revision	Healthy pregnant women in Bangkok, Thailand	68	Simple use of cell phones or radio communication to either make calls or send text messages/short message services (SMS)
Dalton et al. (2018) 31	Cross-sectional	Pregnant women between 10 and 14 weeks	124	Focus groups with pregnant women to define the information needed in the app, in terms of design, literature, content and usability. Control group and later the experimental group.
Chilelli et al. (2014) 15	Theoretical study Revision	Theoretical study of Telemedicine for the care of gestational risk caused by diabetes	n/a	Telemedicine has the potential to revolutionize current methods to manage pregnancy complicated by diabetes, bringing benefits to both patients and healthcare
Whittaker et al. (2012) 32	Longitudinal	Pregnant women and mothers of infants.	100	Analysis with specialists and informal groups with pregnant women to determine the themes.
Felder et al. (2017) 33	Longitudinal	Women at risk of postpartum depression	123	Eight-session Web-based program, with guided mindfulness and yoga practices, cognitive-behavioral strategies, and psychoeducation, consistent with MBCT
Osma et al. (2016) 34	Cross-sectional	English and Spanish speaking women, currently pregnant or within 1 year of giving birth	509	Anonymous online survey to assess access to Information and Communication Technology tools, examine the frequency of searching for health information through online and mobile methods, and identify behaviors related to the access of pregnant and postpartum women to health-related applications.
Forsell et al. (2017) 21	Longitudinal	Pregnant women, with a gestational age between 12-28 weeks and with major depression	42	A modified version of an existing online CBT model was added/adapted to questions closer to the relationship between depression and pregnancy
Nair et al. (2018) 22	Revision	Studies recruited postnatal, perinatal, prenatal, and postpartum women	42 to 852	Traditional phone calls, email and smartphone apps, websites, and chatrooms.
Hantsoo et al. (2017) 35	Cross-sectional	Pregnant women, non-pregnant women and men	312	TTVideo - Patient conducts psychotherapy sessions with the therapist via a web camera, using video chat technology, rather than participating in in-person sessions at the therapist's office. TAComp - The patient completes the psychoeducational modules (usually CBT) through a computer interface. This is complemented with brief face-to-face sessions with a therapist. TOSelf-guided - The patient selects and administers an online program without any interaction with a therapist. The patient completes computer-based exercises at home at their own pace.
Evans et al. (2017) 16	Longitudinal	Women who started the study with scores indicative of depression	318	A nurse-delivered telephone support
Faherty et al. (2017) 23	Longitudinal	Women attending antenatal care at an obstetric clinic affiliated with an urban teaching hospital, serving predominantly minority, racial and ethnic backgrounds, where patients are routinely screened for perinatal depression	36	An application administered by daily surveys (2 questions) and weekly (PHQ-9 and GAD-7). The app measured daily mobility (distance traveled on foot) and travel radius.
Kingston et al. (2017) 36	Cross-sectional	Pregnant women	636	A web-based automated electronic screening intervention group and a paper-based control group
Bischoff et al. (2019) 37	Longitudinal	Pregnant women at the end of the second trimester with of without a psychiatric diagnose.	240	Phone calls and messages from nurses to women who have had a group CBT intervention
Gureje et al. (2015) 38	Longitudinal	Pregnant women with a gestational age between 16 and 28 weeks who tested positive on the Edinburgh Postnatal Depression Scale (EPDS score ≥12)	686	Clinical support and supervision, provided mainly by mobile phone, were provided by general practitioners and psychiatrists. Automated text voice messages, also delivered by cell phones, were used to facilitate patient adherence to clinical appointments and 'homework' tasks.
Green et al. (2019) 24	Longitudinal	Pregnant women	10	Healthy Moms, uses an existing artificial intelligence system called Tess (Zuri in Kenya) to conduct conversations with users. It works by engaging a patient in a conversation through a variety of trusted channels, including text messages (SMS).
Lee et al. (2016) 25	Revision	Pregnant women	n/a	Health information via SMS, psychological intervention, and personalized exercises via audio recordings
Scherer et al. (2014) 39	Longitudinal	Pregnant women between 18 and 32 weeks of gestation diagnosed with preterm delivery	1	An online self-help program for managing anxiety and stress for pregnant women with preterm labor
Gilbert et al. (2015) 40	Longitudinal	Minority pregnant women	724	A smoking cessation program using a cell phone intervention through text messaging and medication use
van den Heuvel et al. (2018) 41	Revision	Studies reporting the use of eHealth during prenatal, perinatal, and postnatal care	1 to 1880	eHealth information and use, lifestyle, pregnancy risk, mental health, telemonitoring and teleconsultation.
Halili et al. (2018) 42	Cross-sectional	Women who were pregnant or gave birth to a baby within six months of the first scheduled focus group	13	The app includes syncing with a newer © Fitbit device, the Charge 2 fitness tracker, which offers sleep, exercise, and diet tracking, as well as daily step counts, as well as mental health and mindfulness techniques that are complemented by content from the application.
Fantinelli et al. (2019) 43	Revision	Telemedicine for Gestational Diabetes Mellitus: an assessment of the psychological dimensions	13	Telemedicine
Hantsoo et al. (2018) 44	Longitudinal	Pregnant women with depressive symptoms (PHQ-9 ≥5) at <32 weeks of gestation	72	A mood tracking and alerting mobile app to improve mental health care delivery in a high-risk obstetric population.
Sondaal et al. (2016) 20	Cross-sectional	1) Pregnant women 2) Pregnant women with HIV	1) 61 2) 40	Unidirectional text messaging
Snaith et al. (2014) 45	Longitudinal	Low-risk nulliparous pregnant women	840	The women received a telephone support intervention

Regarding the information available in the technology, we conducted Content Analysis 29 aiming to identify the strategies used to deliver information, support, or intervention provided. We noticed a prevalent use of techniques and methods based on cognitive-behavioral approaches (44.12%). Regarding the tools available in the technology, we highlight journaling, mentioned five times (7.35%), relaxation exercises, meditation, guided imagery, and psychoeducation, which were mentioned three times (7.35%), along with non-specified techniques based on cognitive-behavioral therapy. Furthermore, strategies including sleep hygiene, *mindfulness* , breathing exercises, feedback, encouragement messages, behavioral activation, journaling, problem-solving, planning of positive activities, and self-care strategies were also listed as techniques and methods based on cognitive-behavioral approaches. The results obtained are presented in Table 2 .

**Table 2:** Content Analysis for Technology Use

Categories	Strategy	Count	Percentage	Total
Cognitive behavioral therapy techniques and methods				44.12%
	Humor records	5	7.35%	
	Relaxation and stress reduction techniques	4	5.88%	
	Meditation	3	4.41%	
	Guided discovery	3	4.41%	
	Exercise not specified	3	4.41%	
	Psychoeducation	3	4.41%	
	Sleep hygiene	2	2.94%	
	Mindfulness	1	1.47%	
	Feedback/ encouragement messages	1	1.47%	
	Behavior activation	1	1.47%	
	Problem resolution	1	1.47%	
	Journaling and Thought Records	1	1.47%	
	Positive activity scheduling	1	1.47%	
	unspecified self-care	1	1.47%	
Social support				14.71%
	General social support	3	4.41%	
	Optional exercises for partners	2	2.94%	
	Support groups	1	1.47%	
	Facilitated health services	1	1.47%	
	Reminders to events, and appointments	1	1.47%	
	Contact the practitioners	1	1.47%	
	Experience reports	1	1.47%	
Mother-baby Link				2.94%
	Connecting to the bay in the womb	1	1.47%	
	Music listening	1	1.47%	
Non-psychological aspects				23.53%
	Healthy diet	4	5.88%	
	Active life	3	4.41%	
	General prenatal information	2	2.94%	
	Infectious disease	1	1.47%	
	Fetal development	1	1.47%	
	Physical changes	1	1.47%	
	Orientation for medical procedures	1	1.47%	
	Glucose level monitoring	1	1.47%	
	General postnatal information	1	1.47%	
	Breastfeeding	1	1.47%	
Substance abuse				4.41%
	Alcohol	1	1.47%	
	Tobacco	1	1.47%	
	Other substances	1	1.47%	
Not cited		5	7.35%	7.35%
Not applicable		2	2.94%	2.94%
Total		68	100%	100%

The period to use the tools ranged from 1 to 40 weeks, depending on the characteristics of the intervention. On average the technology use lasted around 13 weeks. The interventions similar to the ones proposed by Carissoli et al. (2016) 13 were shorter. Specifically, these authors investigated the user experience with a mobile app for the well-being of pregnant women, during one week, aiming to bring awareness to their affective states, besides also teaching them strategies to cope with anxiety and stress. We also found studies, like the one conducted by Gureje et al. (2015), 38 which were extended – initially beginning with eight weekly sessions but including additional sessions during pregnancy on demand as well as an intervention six weeks after the birth of the child, depending on the levels of depressive symptoms notice in the mother during postpartum. In this case, specifically, the support and the clinical supervision were delivered mainly via mobile phone by doctors and psychiatrists. Besides this, they sent patients text and voice messages automatically, through mobile phones, in order to: (a) improve the compliance of patients with clinical appointments and (b) follow up on their recommendations. Additional studies applied telemedicine to monitor women throughout their pregnancies. 16 45

In our analysis, we investigated whether the objectives of the studies included the study of technology usage, specifically regarding certain pathologies, or co-morbidities occurring during pregnancy. As a result, we found that 59.3% (16 papers) described the usage of technology related to treat specific pathologies. Also, 40.7% (11 papers) did not mention such objective. The pathologies mentioned are listed in Table 3 .

**Table 3:** Mental illnesses and pathologies

Pathologies	Frequency	Percentage
Depression	12	40.0
Anxiety	1	3.3
Diabetes	1	3.3
Stress	1	3.3
High risk pregnancy	1	3.3
HIV	1	3.3
Pre-term birth	1	3.3
Tobacco use	1	3.3
Not applicable	11	36.7
Total	30	100.0

## Discussion

By providing an overview of the literature, this study aimed to evaluate the applicability, the benefits, and limitation related to technology usage in prenatal care, specifically regarding mental health. This review shows that the interventions have a wide range of applications in what regards prenatal care. The categorical analysis indicates that general aspects beyond psychological factors are also covered, including clinical factors, obstetrics, and socioeconomic aspects. Thus, we noticed that the multidisciplinary approaches are emphasized in the studies analyzed.

For example, Oliveira-Ciabati et al. (2017) 46 identified that a short text message service is potentially useful for improving coverage of antenatal health practices, including syphilis and HIV testing. However, only a fifth of eligible women showed interest in and applied to join this project. Thus, the authors recognize the importance of further encouraging participants to use such tools, realizing that there are still several obstacles that need to be overcome in order to be successful in expanding the proposed interventions.

Our results show that most studies were carried out in developed countries. Hence, it is crucial to further discuss the possibilities to implement healthcare strategies driven by technology also in low- and middle-income countries, seeking to identify their specific characteristics, resources, contextual aspects and patients' needs. According to a report published by the Brazilian Institute of Geography and Statistics (IBGE) in 2018, 47 the Internet was used in 79.1% of Brazilian homes, but 99.2% of the homes had access to the Internet through a mobile phone. When compared to 2017, the Internet usage grew (74.9%). Still, it is important to highlight the inequalities in what regards access to Information and Communication Technologies (ICTs) seeking to provide a more democratic use for such services.

In what regards the information available through technology considering the usage of cognitive-behavioral strategies, the literature provides evidence that support the efficacy of cognitive-behavioral therapies, especially from the third-wave cognitive behavioral therapy, to improve the patients' health outcomes as well as symptoms of depression, anxiety, and overall quality of life after treatment. Furthermore, the results indicate a significant efficacy obtained when such treatments are compared to control treatments for active management of depression and anxiety. 48

Because we selected publications based on a set of pre-defined descriptors, this review is not comprehensive. Despite the limitations of this study, the results obtained contribute to augment the discussion around the usage of technology for healthcare, and specifically for Psychology. In this context, it is relevant to consider technology use for mental health along with quality of care, data security, opportunities involved, efficacy, efficiency, and patient focus. To facilitate the implementation and usage of technology strategies, policy makers must consider the international legislation to implement and validate this new format in the delivery of health services. 41

## Conclusion

Technology usage has been vastly explored for health interventions. Regarding their usage for mental health during pregnancy, this analysis of the literature suggests a growth in its development, as well as the need to further develop and study it in Brazil and Latin America, since we could not find any articles published in such countries. Still, these practices have a vast array of applications, for multiple purposes, even when focusing on prenatal care. Overall, we noticed a large adoption of apps and smartphones, as well as SMS. Also, technology has shown to be relevant in promoting and facilitating psychological interventions, in special using techniques and methods that adopt cognitive behavioral approaches. The duration of the interventions varied substantially, indicating diverse possibilities of use for tools and interventions in what regards screening, treating, or monitoring mental health. We highlight thus, that the use of technology for mental health among pregnant women is still being investigated as a potential mechanism for healthcare delivery. In this context, despite the limitations involved in this scientific study, we notice the need to further investigate, research and develop this domain, to explore interventions that could improve the quality of prenatal care, contributing to protect women's and children's health.
